# Activity of the inferior parietal cortex is modulated by visual feedback delay in the robot hand illusion

**DOI:** 10.1038/s41598-019-46527-8

**Published:** 2019-07-11

**Authors:** Mohamad Arif Fahmi Bin Ismail, Sotaro Shimada

**Affiliations:** 1 Graduate School of Science and Technology, Meiji University, Kanagawa, Japan; 2 School of Science and Technology, Meiji University, Kanagawa, Japan

**Keywords:** Consciousness, Perception

## Abstract

The robot hand illusion (RoHI) is the perception of self-ownership and self-agency of a virtual (robot) hand that moves consistently with one’s own. The phenomenon shows that self-attribution can be established via temporal integration of visual and movement information. Our previous study showed that participants felt significantly greater RoHI (sense of self-ownership and sense of self-agency) when visuomotor temporal discrepancies were less than 200 ms. A weaker RoHI effect (sense of self-agency only) was observed when temporal discrepancies were between 300 and 500 ms. Here, we used functional near-infrared spectroscopy (fNIRS) to investigate brain activity associated with the RoHI under different visual feedback delays (100 ms, 400 ms, 700 ms). We found that the angular and supramarginal gyri exhibited significant activation in the 100-ms feedback condition. ANOVA indicated a significant difference between the 100-ms condition and the other conditions (*p* < 0.01). These results demonstrate that activity in the posterior parietal cortex was modulated by the delay between the motor command and the visual feedback of the virtual hand movements. Thus, we propose that the inferior parietal cortex is essential for integrating motor and visual information to distinguish one’s own body from others.

## Introduction

Most people have sense of strong control over their voluntary actions and can also distinguish between their own body and those of others. This implies that self-attribution comprises two sensory components: sense of agency (SoA) and sense of ownership (SoO)^1,2^. The SoA is the sense of authorship for a given action, which is a subjective feeling of control over one’s actions and their outcomes. The SoO is the conscious awareness that one’s body belongs to oneself.

In the rubber hand illusion (RHI), observers feel the SoO toward a fake hand (rubber hand) when their hand and the rubber hand are stroked simultaneously, while the observer does not feel that the rubber hand is their own hand when they receive visual and tactile information from the rubber hand asynchronously^3–6^. In addition, the result showed a significant difference in proprioceptive drift toward the rubber hand in synchronous condition in congruent posture. The proprioceptive drift is defined as the drift of the perceived location of one’s own hand before and after the stimulation. Usually, the drift occurs toward the rubber hand when the RHI takes place. The participants felt more strongly that the rubber hand is their own when the stimuli were applied on their hand and the rubber hand synchronously compared to the asynchronous stimulation^5^. Studies have addressed the time window for this self-attribution in RHI, showing that recognition was attenuated when visual information was delayed longer than 200–300 ms^7,8^.

While the integration of visual and tactile information, and thus the SoO, have been investigated in RHI studies, RHI have been expanded to investigate not only the SoO but also the SoA using a ‘moving rubber hand’ paradigm^9,10^. Kalckert & Ehrsson modified the RHI experimental paradigm to address the active movement, passive movement and visuotactile stimulation on the rubber hand. The participant’s index finger was connected to the index finger of a fake hand. The participant was instructed to lifts their index finger, so that they can feel the SoA of that finger movement of the fake hand. In the synchronous active condition, the participant felt both the SoO and SoA of the fake hand stronger than in other conditions. In addition, the participants also felt greater SoA when they moved their own hand (active condition) compared to when the experimenter moved the participant’s hand (passive condition). In addition, the virtual hand illusion using the same paradigm with the RHI also showed that the participants felt both the SoO and the SoA toward the virtual hand that is generated by computer graphics (CG) as their own hand in the synchronous condition compared to the asynchronous condition^11,12^.

In this study, we focused on the integration of visual and motor information on the fake hand, which is called as the robot hand illusion (RoHI)^13–15^. The RoHI is an illusion in which a virtual (robot) hand is perceived as one’s own hand when it moves consistently with one’s hand motions. Our previous study showed that attenuation of self-attribution toward a virtual hand occurred when visual feedback was delayed longer than 200 ms^13^. The present study aimed to further investigate the neural mechanism of the SoO and the SoA when experiencing the RoHI.

The neural mechanism that underlies self-attribution is still unclear. Several neuroimaging studies have used the RHI to show that the premotor cortex is related with the SoO^16–19^. Ehrsson *et al*. also suggested that activity in the intraparietal cortex preceding the onset of the RHI, which likely plays a role in the continued multisensory integration of stimuli with respect to the hand^16^. Furthermore, studies have found that the intraparietal region is related to the integration of visual and tactile information that is associated with the RHI^17,18,20–22^. The neural correlates of SoA have been also suggested to be related to activity in the inferior parietal lobule (IPL)^23–26^.

The above-mentioned studies have investigated the multisensory integration for the self-body senses, namely the SoO and the SoA, and the neural networks correlated with the self-attribution. The present study systematically investigated self-attribution in the RoHI under delayed visual feedback. We focused on the investigation of the temporal binding between visual and motor information and conducted a neuroimaging experiment by using functional near-infrared spectroscopy (fNIRS). We predicted that the temporal window needed for visual and motor integration in the RoHI is less than 200–300 ms like in the RHI, and that the IPL is essential to establish both the SoO and the SoA.

## Results

### Questionnaires

**Figure 1:**
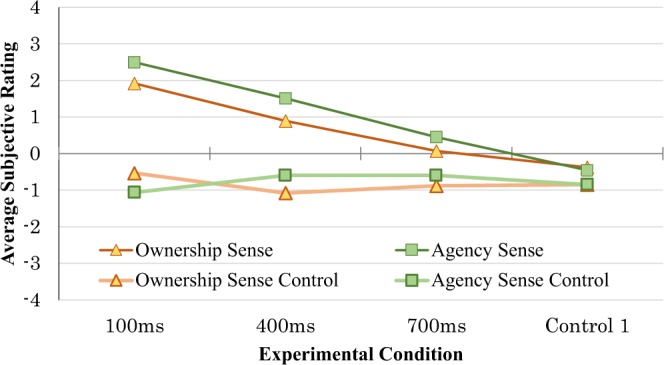
Average ownership sense, ownership sense control, agency sense, and agency sense control related questions.

The average scores for questionnaire items related to the SoO (items 1–4) and those related to the SoA (items 9–12) are shown in Fig. 1 for all participants. Participant ratings were higher in both measures for the 100-ms delayed visual feedback condition than for the other conditions. The Kolmogorov-Smirnov test indicates that the data follow the normal distribution (SoO, D(64) = 0.144, *p* > 0.05; SoA, D(64) = 0.120, *p* > 0.05; ownership sense control, D(64) = 0.066, *p* > 0.05; agency sense control, D(64) = 0.91, *p* > 0.05).

**Figure 2:**
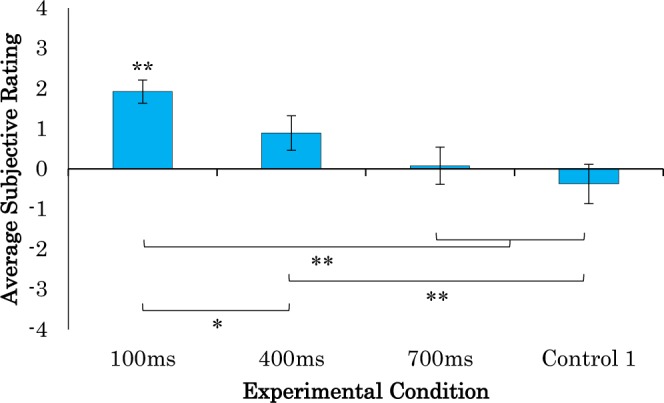
Average subjective ratings for sense of ownership-related questions. **p < 0.01, *p < 0.05.

**Figure 3:**
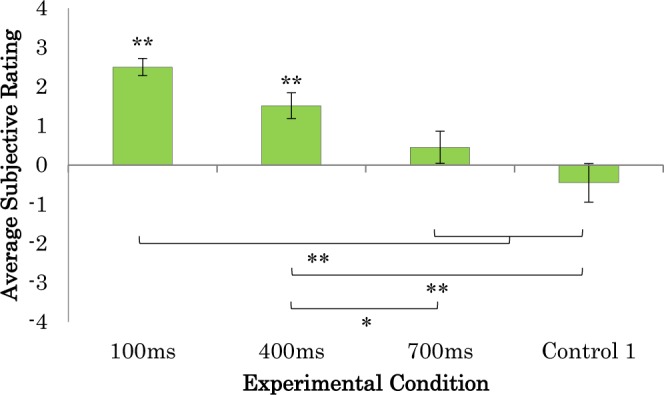
Average subjective ratings for sense of agency-related questions. **p < 0.01, *p < 0.05.

The subjective ratings are shown in more detail in Figs 2 and 3. We performed a 4 × 2 two-way ANOVA with delay (100 ms, 400 ms, 700 ms, and control 1; see Methods) and sense (ownership and agency) as factors. The results showed a significant main effect of delay (*F*_(3, 120)_ = 17.248, *p* < 0.01, η^2^ = 0.657), but no significant main effect of sense (*F*_(1,120)_ = 1.860, *p* > 0.1, η^2^ = 0.125) or any interaction between the two (*F*_(3,120)_ = 0.341, *p* > 0.1, η^2^ = 0.092). For both the SoO and the SoA, subsequent analyses (Tukey’s honestly significant difference, HSD) showed that the questionnaire scores for the 100-ms condition were significantly greater than those for the 700-ms and control conditions (*p* < 0.01), and those for the 400-ms condition were significantly greater than those for the control-1 condition (*p* < 0.01). Additionally, the SoO was significantly greater in the 100-ms condition than in the 400-ms condition (*p* < 0.05), and the SoA was greater in the 400-ms condition than in the 700-ms condition (*p* < 0.05). The post-hoc power analysis showed that we have enough power (96.1%) for this analysis (16 samples for each condition).

A one-way (delay) ANOVA was applied separately to the ownership-sense control and the agency-sense control items. For both items, the results showed no significant main effect of delay (ownership sense control, *F*_(3, 60)_ = 0.525, *p* > 0.1, η^2^ = 0.162; agency sense control, *F*_(3, 60)_ = 0.432, *p* > 0.1, η^2^ = 0.147; Fig. 1).

### fNIRS results

**Figure 4:**
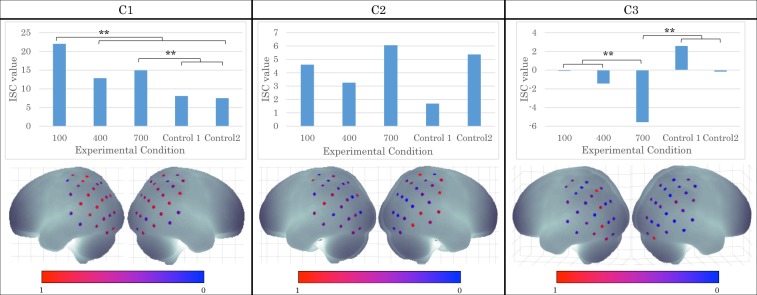
The result of CCA analysis and ISC analysis of each component (C1, C2, C3).

Using correlated component analysis (CCA, see Methods), we analysed the differential effects among correlated components to determine which experimental conditions affect neural activity. Each component of the correlated activity has different spatial distributions on the scalp. Figure 4 shows the top three components (first component, C1; second component, C2; third component, C3). The inter-subject correlation (ISC, see Methods) was calculated for each component and condition, which then submitted to one-way ANOVA (100–700 ms delay, control 1 and 2) to determine the conditional effect.

The first CCA component was pronounced over the angular gyrus and the supramarginal gyrus in both hemispheres, the visual association cortex in the left hemisphere, and the somatosensory association cortex in the right hemisphere. ANOVA for this component showed a significant main effect of delay (*F*_(4,75)_ = 3.466, *p* < 0.05, η^2^ = 0.430). Subsequent analyses (Tukey HSD) revealed that ISC for the 100-ms condition was significantly greater than those for the other conditions (*p* < 0.01). Similarly, ISC for the 700-ms condition was significantly greater than those for the control conditions (*p* < 0.01).

The second component was strong over the somatosensory cortex and the supramarginal gyrus in both hemispheres and the superior middle temporal gyrus in the right hemisphere. The result showed no significant main effect of delay (*F*_(4,75)_ = 1.086, *p* > 0.1, η^2^ = 0.240).

The third component was strong over the visual association cortex in both hemispheres, the somatosensory association cortex and the angular gyrus in the left hemisphere. ANOVA showed a significant main effect of delay (*F*_(4,75)_ = 2.519, *p* < 0.05, η^2^ = 0.367). Subsequent analyses revealed that ISC for the 700-ms condition was significantly weaker than those for the other conditions (*p* < 0.01).

## Discussion

The questionnaire results in this experiment showed that the participants felt the sense of ownership only when visual feedback occurred within 100 ms of their movement, and the sense of agency when it occurred within 400 ms (see Figs 2 and 3). However, the participants rated the 700 ms condition with no significant difference compared to 0 (neutral) for both the SoO and the SoA questionnaires. In addition, the angular gyrus and the supramarginal gyrus showed significant activation in the 100-ms delayed visual feedback condition, which was significantly different from the other conditions (p < 0.01). This result was consistent with our previous findings that greater SoO and SoA occurred when the visual feedback was delivered within less than 200 ms delay and that weak SoA occurred with delays between 300 ms and 500 ms^13^. It was also shown that there was no significant SoO or SoA when visual feedback was delayed more than 500 ms. Several studies have shown that the time window for visual and tactile integration is 200–300 ms in the case of RHI^7,8^. Further, other studies of auditory and visual integration have also indicated that the time window for integrating these senses was less than 200 ms^27–29^. However, according to Costantini *et al*., the temporal limit on the RHI depends on individual’s perceptual temporal resolution^30^. The participants need to judge the simultaneity between visual and the tactile stimuli. In their study, the results showed that the average of the temporal binding window was 211 ± 59.9 ms, which are less than 300 ms. Then, the participant underwent the RHI stimulation and felt a significantly greater SoO in the synchronous condition compared to the asynchronous condition where the individual size of temporal binding window was used as the visual feedback delay in the asynchronous condition. This study is consistent with our finding where multisensory integration for self-attribution in RoHI occurs only when the two sensations occur within 300 ms of each other.

In this experiment, we focused on the RoHI to investigate brain activity related to the SoO and SoA. The feeling of body ownership has been suggested to be related to activity in the posterior parietal cortex and the intraparietal sulcus (PPC/IPS) as well as in the premotor cortex^31–33^. According to Makin *et al*. the PPC seems to integrate multisensory information with respect to the rubber hand^31^. Moreover, Ehrsson *et al*. identified activity within the anterior part of the IPS preceding the onset of the RHI, which likely plays a role on the continued multisensory integration with respect to the hand^32^. Linear relationship between proprioceptive drift and brain activity in the supramarginal gyrus is also reported^18^. This finding indicates that the SoO has been associated with activity in the supramarginal gyrus. These findings are consistent with our results, in which the first CCA component showed that the angular gyrus and the supramarginal gyrus activated in the 100 ms condition compared to the other conditions.

Additionally, the SoA has also been associated with activity in the inferior parietal lobule (IPL)^25,34,35^. According to Renes *et al*., the IPL showed more activation when the outcome matched with the participant’s goal and the participant felt greater SoA than when not^36^. This finding is consistent with our results that the first and third CCA components, which both include the IPL regions, showed more activation in the 100 ms delay condition compared to the other, especially to the 700 ms, delay conditions. The participants also rated the SoA score lower in the 700 ms condition than the other conditions. These indicate that the activation in the IPL is associated with a feeling of agency toward a robot hand. To sum, our result showed that the IPL was activated when participants felt both SoO and SoA in the 100-ms delayed visual feedback condition. We postulate that the IPL is an essential area for multisensory integration regarding the self-body.

## Methods

### Participants

Sixteen healthy students (all male; aged 22.5 ± 1.0 years) who were naive to the purpose of the study were recruited for the experiment. Thirteen participants were right-handed and three were left-handed and all had normal or corrected-to-normal vision. All participants provided written informed consent. The experiment was approved by the ethics committee of the School of Science and Technology, Meiji University (No. 08562), and was conducted according to the principles and guidelines of the Declaration of Helsinki.

### Procedure

**Figure 5:**
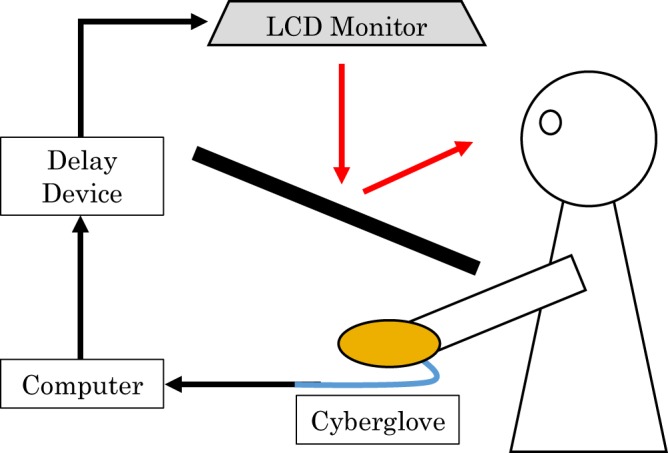
Experimental setup. The participants could observe the delayed virtual hand consistently like their own hand movement.

The participants were asked to sit at a table with a mirror and put their right palm facing down. The participants were not able to see their own right hand directly (see Fig. 5). Using the Cyberglove system (Cyberglove, CyberGlove Systems LLC, San Jose, California), the participants were instructed to move their hand to record their hand movement. An image of a virtual hand, which was generated using recorded data, was presented on a liquid-crystal display monitor (LMD-232W, SONY, Tokyo, Japan) through the reflection of the mirror. Visual feedback delay was constructed using a hardware device (EDS3305, Eletex, Osaka, Japan). Three delay conditions (100, 400 and 700 ms) and two control conditions (control 1, the subject observed the video of the virtual hand moved without moving their own hand; control 2, the subject moved their hand without observing the virtual hand) for hand-opening and hand-closing movements was tested for each participant. Each trial lasted 30 s (5 s for pre-rest, 10 s for task, and 15 s for post-rest), which was repeated 6 times. We recorded fNIRS data for each delay conditions. The order of the delay conditions was pseudo-random and counterbalanced across participants.

After completing each condition, participants completed a 16-item questionnaire identical to that used in previous studies except in control 2 condition because participants do not observe any image of hand^9,13^, in either the original English or in Japanese. They reported their subjective experience on a 7-point Likert-like scale ranging from −3 (totally disagree) to +3 (totally agree), with 0 indicating neither agreement nor disagreement (uncertain). Four statements referred to the feeling of ownership (e.g., “I felt as if I was looking at my own hand”), and four statements described sensations related to agency (e.g., “I felt as if I was causing the movement I saw”). The remaining eight statements were control statements which is describe the fake question of the senses; four describing ownership and the other four describing agency (e.g., “I felt as if I had more than one right hand” and “It seemed as if the hand image had a will of its own”).

### fNIRS data acquisition

The fNIRS data were recorded using a multichannel fNIRS unit operating at 780-nm, 805-nm, and 830-nm wavelengths (OMM-3000, Shimadzu, Japan) at a sampling frequency of 10 Hz. Oxy-haemoglobin (oxyHb), deoxy-haemoglobin (deoxyHb), and total haemoglobin (totalHb) concentrations were recorded in the temporal, parietal, and occipital lobes. The optode locations were recorded using a 3D magnetic space digitizer (Fastrak, Polhemus, USA) to estimate the anatomical brain region beneath the fNIRS channels. To determine the correspondence between the measured position data and the fNIRS channels, we used the probabilistic spatial registration method^37^ to generate a probabilistic mapping between each fNIRS channel and its corresponding anatomical brain region. This was then used for interpreting fNIRS-activation data.

### fNIRS data analysis

The change in oxyHb is considered the main parameter that changes with regional cerebral blood flow. A 2-Hz low-pass filter and a 0.2-Hz high-pass filter were applied to the fNIRS data. CCA was applied to the fNIRS signal for each condition to extract the common signal that was contained in all conditions^38–40^ and to analyze up to the third component. In CCA, we find weight vector for the data to maximize the correlation coefficient between experiment conditions. The signals commonly contained in different conditions for each channel was extracted through CCA. Additionally, the brain region involved in the component was determined using a forward model, A = RW(WTRW) − 1^41,42^, using weight (W) that produced from CCA and data covariance matrix (R). Each component has been normalized so that the maximum becomes 1. In order to compare the degree to which cortical activity was correlated among the conditions, we took the sum of the averaged correlations for every pair of participants for each component (known as ISC)^40^. It extracts the common brain activity among the subjects performing the same task. We used general linear model (GLM) which is an analysis method for statistically examining how much the observed signal (of one participant) can be fitted with a model called design matrix (another participant’s signal). Comparisons across conditions were performed using analysis of variance (ANOVA).
